# Calcification of the visceral aorta and celiac trunk is associated with renal and allograft outcomes after deceased donor liver transplantation

**DOI:** 10.1007/s00261-022-03629-8

**Published:** 2022-11-28

**Authors:** Robert Siepmann, Philipp Bruners, Sven Arke Lang, Jan Bednarsch, Iakovos Amygdalos, Katharina Joechle, Federico Pedersoli, Sebastian Keil, Peter Isfort, Tom Florian Ulmer, Christiane Kuhl, Ulf Peter Neumann, Franziska Alexandra Meister, Zoltan Czigany

**Affiliations:** 1 Department of Diagnostic and Interventional Radiology, University Hospital RWTH Aachen, Aachen, Germany; 2 Department of Surgery and Transplantation, University Hospital RWTH Aachen, Pauwelsstrasse 30, 52074 Aachen, Germany; 3 Department of Surgery, Maastricht University Medical Centre (MUMC), Maastricht, The Netherlands; 4 Department of Surgery, Campus Charité Mitte | Campus Virchow-Klinikum, Charité-Universitätsmedizin Berlin, Berlin, Germany

**Keywords:** Liver transplantation, Aortic calcification, Celiac trunk calcification, Agatston calcium score

## Abstract

**Purpose:**

Atherosclerosis affects clinical outcomes in the setting of major surgery. Here we aimed to investigate the prognostic role of visceral aortic (VAC), extended visceral aortic (VAC+), and celiac artery calcification (CAC) in the assessment of short- and long-term outcomes following deceased donor orthotopic liver transplantation (OLT) in a western European cohort.

**Methods:**

We retrospectively analyzed the data of 281 consecutive recipients who underwent OLT at a German university medical center (05/2010–03/2020). The parameters VAC, VAC+, or CAC were evaluated by preoperative computed tomography-based calcium quantification according to the Agatston score.

**Results:**

Significant VAC or CAC were associated with impaired postoperative renal function (*p* = 0.0016; *p* = 0.0211). Patients with VAC suffered more frequently from early allograft dysfunction (EAD) (38 vs 26%, *p* = 0.031), while CAC was associated with higher estimated procedural costs (*p* = 0.049). In the multivariate logistic regression analysis, VAC was identified as an independent predictor of EAD (2.387 OR, 1.290–4.418 CI, *p* = 0.006). Concerning long-term graft and patient survival, no significant difference was found, even though patients with calcification showed a tendency towards lower 5-year survival compared to those without (VAC: 65 vs 73%, *p* = 0.217; CAC: 52 vs 72%, *p* = 0.105). VAC+ failed to provide an additional prognostic value compared to VAC.

**Conclusion:**

This is the first clinical report to show the prognostic role of VAC/CAC in the setting of deceased donor OLT with a particular value in the perioperative phase. Further studies are warranted to validate these findings.

**Graphical abstract:**

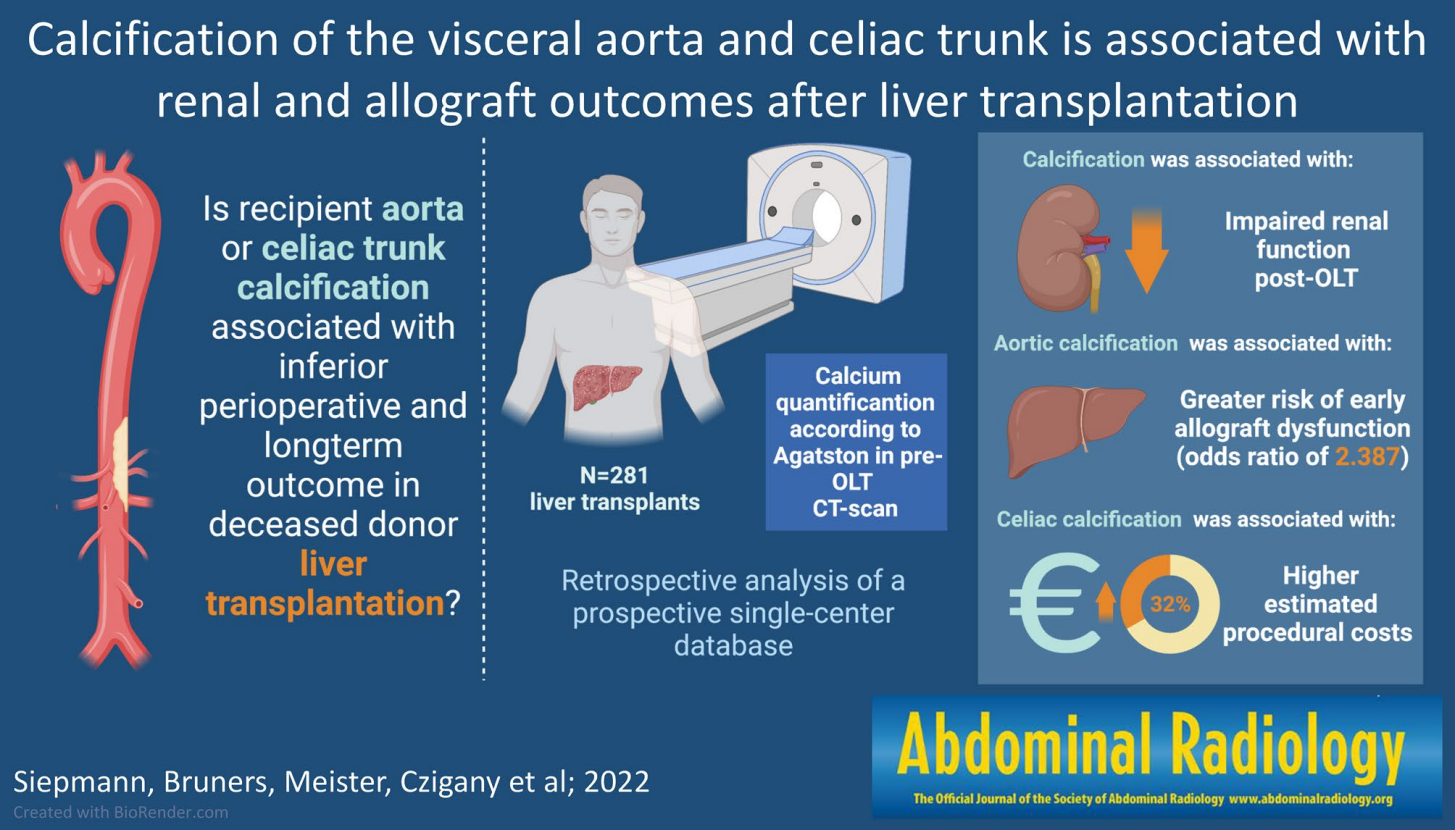

*CT* computed tomography, *OLT* orthotopic liver transplantation

**Supplementary Information:**

The online version contains supplementary material available at 10.1007/s00261-022-03629-8.

## Introduction

Atherosclerosis has become a rising issue in patients suffering from end-stage liver disease (ESLD). Patients with advanced cirrhosis present with a low systemic vascular resistance combined with a reduced arterial compliance [1]. Cholesterol metabolisms and coagulation factor production are among the main functions of the liver which are impaired in cirrhosis. Therefore, up to 40% of patients with ESLD present with significant atherosclerotic disease prior to orthotopic liver transplantation (OLT) [1]. These patients are frequently suffering from visceral aortic calcification (VAC) which is known to be associated with coronary artery disease and cardiovascular events including myocardial infarction and stroke [2]. On this account, it is of utmost clinical importance to appropriately estimate the cardiovascular risk of all patients being considered for OLT, as cardiovascular complications are a major cause of morbidity and death, not only in the perioperative time but especially during the first year post-OLT [3]. Nevertheless, there is no international consensus on the preferred pre-OLT screening to detect significant coronary artery and other vascular disease [4].

Particularly, in times of critical organ shortage and consecutive rise in the use of marginal allografts from extended criteria donors (ECD), an optimal clinical risk assessment and outcome prediction in the setting of OLT is of utmost relevance [5, 6]. In this context, various predictive scores have been developed to assess perioperative risk in the setting of OLT over the last decades [7, 8]. These include well-established scores such as Balance of Risk (BAR) [9], Survival Outcomes Following liver Transplantation (SOFT) [10], and the Donor Risk Index (DRI) [11, 12]. The Liver Graft Assessment Following Transplantation (L-GrAFT) [13] and Early Allograft Failure Simplified Estimation (EASE) [14] ratings, which concentrate on early allograft dysfunction, have also been developed recently. In addition, few studies focusing on individual parameters such as postoperative platelet counts [15] or body mass index (BMI) [16] could demonstrate an association between these parameters and perioperative outcomes. However, due to the fact that data on significant vessel calcification are not routinely reported in major multi-center or multi-national registries, none of the previous risk-assessment models and scores contain vascular calcification as a relevant risk factor.

While incidence of VAC and its impact on postoperative outcome have been reported in living- and deceased donor kidney transplantation by various groups [17, 18], its role in predicting outcome in OLT remains largely unexplored [19, 20]. However, Imaoka et al. recently showed a strong association between VAC and impaired overall survival in patients undergoing OLT [19, 20]. Using the scoring system developed by Agatston, VAC can easily be quantified on abdominal CT scans which are routinely available in most OLT candidates [21].

The aim of this study was to comprehensively investigate the incidence and role of visceral arterial calcification in predicting perioperative and long-term outcomes in a European single-center cohort of patients undergoing OLT after deceased donation.

## Materials and methods

### Study population and ethics

Data from all consecutive patients who underwent deceased donor OLT between 05/2010 and 03/2020 at the University Hospital RWTH Aachen (UH-RWTH), Aachen, Germany, were taken into consideration for inclusion in this retrospective study. All patients with a plain abdominal CT scan not older than 12 months prior to surgery were included. Patients who received living-related or split liver transplantation as well as patients undergoing re-OLT were excluded. The RWTH Aachen University Institutional Review Board (EK 341/21) authorized this study, which follows the principles of the current edition of the Declaration of Helsinki, as well as the Declaration of Istanbul and the good clinical practice (ICH-GCP) recommendations. The local IRB waived informed consent due to the retrospective study design and collection of routine clinical data.

### CT Imaging and quantification of calcification

**Fig. 1:**
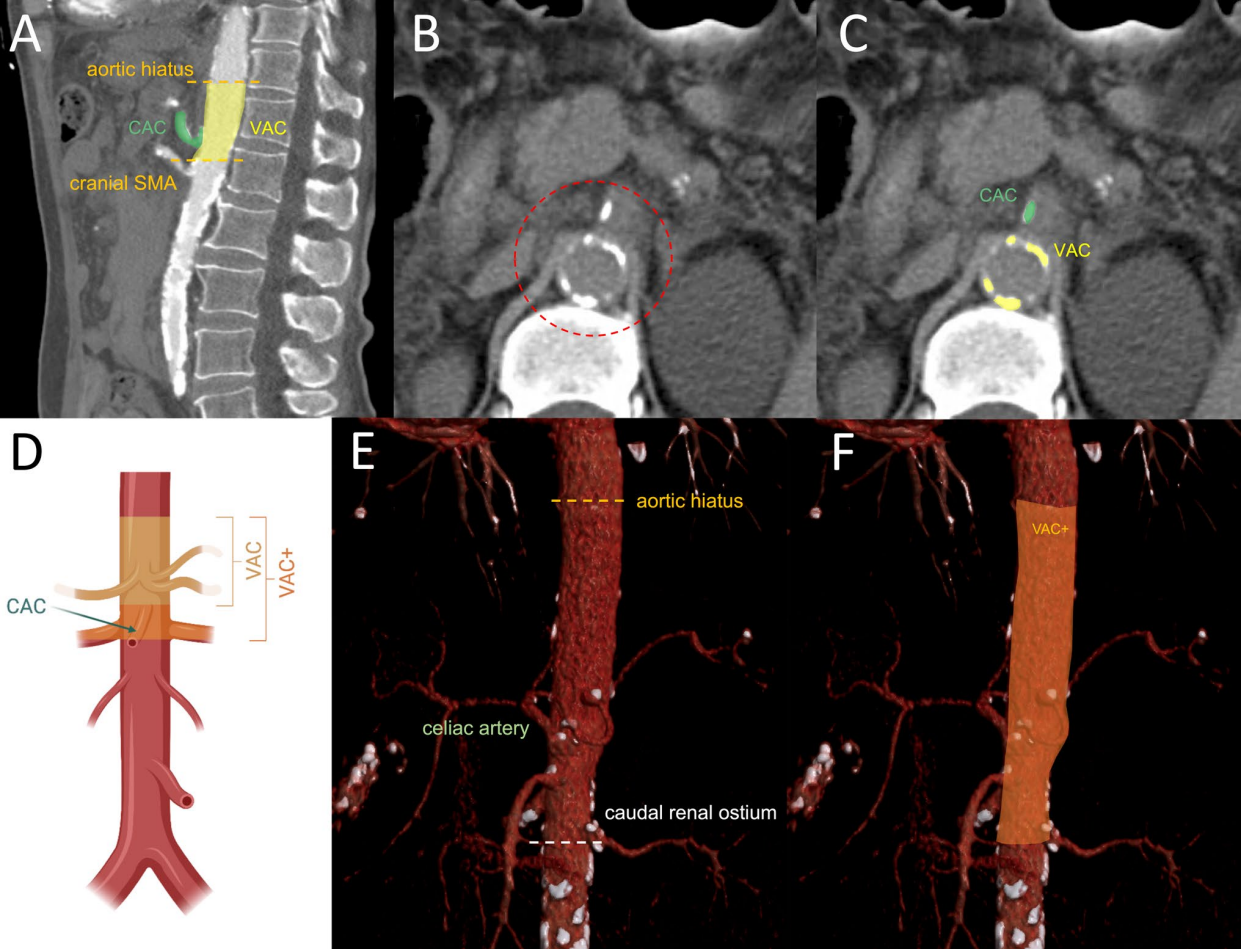
Visceral calcium scoring. **a** Sagittal image and anatomical definition of VAC and CAC. **b**, **c** Axial images showing calcification of the visceral aorta and celiac artery. **d** Schematic representation of the assessed parameters, VAC, VAC+ and CAC. **e**, **f** 3D Reconstruction of the anatomical landmarks for the definition of extended visceral aortic calcification or VAC+ . *CAC* celiac artery calcification, *VAC* visceral aortic calcification, *VAC+*  extended visceral aortic calcification including the renal artery ostia

Technical parameters for CT imaging were chosen as following: 128-section CT scan (SOMATOM Definition Flash, Siemens Healthcare, Erlangen, Germany) with 128 × 0.6 mm section collimation, a gantry rotation time of 0.5 s, a tube potential of 120 kV or a 40-section CT scan (SOMATOM Definition AS, Siemens Healthcare, Erlangen, Germany). 5/4 mm and 1/7 mm axial reconstructions were performed. Separate calcium scoring of the proximal, visceral aorta (VA) and of the celiac artery (CA) were performed according to the Agatston method, as shown in Fig. 1. Briefly, abdominal plain non-enhanced CT scans were used to identify the above-mentioned vascular structures. The cranial beginning of the visceral aortic segment was anatomically defined by the upper margin of the aortic hiatus. More precisely, the first axial slice from the direction of the thoracic aorta in which the diaphragmatic crura visibly come in contact with the descending aorta was defined as the upper border of the visceral aortic segment. The caudal border was defined by the cranial beginning of the superior mesenteric artery (SMA) for VAC and by the caudal margin of the lowest renal artery ostium for extended visceral aortic calcification (VAC+) (see also Fig. 1).

Calcification volume was calculated in a semiautomatic fashion with the SyngoVia software module (Siemens Healthineers, Erlangen, Germany) using a weighted density score based on the highest radiodensity of a given calcified plaque multiplied by its area with a cutoff value of 130 Hounsfields units (HU). Quantification of the calcified plaques expanding from aortic ostia into the visceral vessels was performed by excluding the part of the plaque located outside the aortic circumference. All measurements were carried out by an experienced radiologist in training and were validated by a senior staff radiologist.

### Data collection and follow-up

Clinical data were collected from prospective institutional database and analyzed retrospectively. The definitions of the German Medical Chamber were used to define ECD^6^. All of the criteria, scores, and classifications utilized in this paper have been detailed in earlier studies of our group and by others (early allograft dysfunction-EAD [22], OLT risk scores [5, 10, 15, 23], Clavien–Dindo classification—CD, and the Comprehensive Complication Index—CCI [24, 25], calculations of length of hospitalization [26], estimated procedural costs [27] and peri- and postoperative transfusions [28]). Our transplantation outpatient clinic as well as the responsible general practitioner and/or community-based hepatologist provided all follow‐up data used in this study. As previously described, OLT was carried out utilizing a standardized technique of total cava replacement [16, 29]. The standard immunosuppression regime consisted of basiliximab, tacrolimus, mycophenolatemofetil, and corticosteroids for all patients [26].

### Study endpoints and statistical analysis

Post-OLT new-onset impaired renal function was chosen as the primary endpoint for the study. EAD, estimated procedural costs, postoperative 90-day mortality and major morbidity (Clavien–Dindo-CD ≥ 3b), length of ICU and hospital stay and 5-year graft and patient survival represented the secondary endpoints.

Data were reported as median (interquartile range-IQR). In case of categorical variables, absolute and relative frequencies were reported. For analysis of categorical data, the Χ^2^ test and the Fisher exact test were used. For comparison of continuous variables, the Mann–Whitney *U* test was applied. In case of continuous data with multiple measurements over time, the 2‐way ANOVA analysis of variance with Bonferroni post-hoc test was utilized. To stratify the cohort in low- and high-risk sub-cohorts, cutoffs of the calcifications scores were defined using the area under the receiver operating characteristic curve (AUROC) analysis and Youden-Index using “new-onset renal failure” as state variable, as described by our group before [5, 15]. To further evaluate the relationship between calcification levels and outcomes, uni- and multivariate logistic regression analyses were performed. The Kaplan–Meier method was used to visualize patient and graft survival. Survival data was analyzed with the log-rank test. All *p* values < 0.05 were considered statistically significant. Statistical analysis has been performed using SPSS Statistics v24 (IBM Corp., Armonk, NY).

## Results

### Recipient and donor characteristics

In accordance with the above-mentioned exclusion criteria, an overall of 281 out of 429 patients were included in the study. The median age of OLT recipients was 58 [51–63] years (Table 1). The majority of recipients were male (198; (70%)), and the most common indication for OLT was hepatocellular carcinoma (32%), closely followed by alcoholic liver cirrhosis (29%) (Table 1). The median pre-OLT laboratory Model of End-stage Liver Disease (labMELD) score [30] was 17 [10–27]. The median age of the donors was 58 [49–68] (Table 1). Some 145 donors were male and 136 were female (Table 1). The leading causes of death in the donor were cerebrovascular accidents (58%), anoxia (23%) followed by trauma (13%). Sixty-one percent of the donors met ECD criteria (Table 1). Further information on donor and recipient characteristics are shown in Table 1.

**Table 1:** Donor- and recipient characteristics

Characteristics	All patients	VAC > 100 mm^3^		CAC > 50 mm^3^		*p* value
	*n* = 281	Yes *n* = 81	No *n* = 200	Yes *n* = 19	No *n* = 262	
Donor characteristics						
Gender (F:M)	136 (48):145 (52)	37 (46):44 (54)	99 (49):101 (51)	11 (58):8 (42)	125 (48):137(52)	0.327/0.268
Age (years)	58 [49–68]	59 [51–71]	57 [48–66]	63 [53–68]	57 [48–68]	0.189/0.255
BMI	28 [25–31]	28 [25–31]	28 [25–31]	29 [26–34]	28 [25–31]	0.500/0.072
Donor Risk Index^a^	1.77 [1.55–1.99]	1.90 [1.61–2.05]	1.72 [1.51–1.98]	1.94 [1.65–1.99]	1.77 [1.53–1.99]	0.087/0.601
Donor cause of death						
CVA	164 (58)	47 (58)	117 (59)	9 (47)	155 (59)	0.523/0.221
Anoxia	64 (23)	18 (22)	46 (23)	4 (21)	60 (23)	0.511/0.557
Trauma	36(13)	12 (15)	24 (12)	3 (16)	33 (13)	0.323/0.450
Other	17 (6)	4 (5)	13 (6)	3 (16)	14 (5)	0.426/0.097
Extended Criteria Donor Allografts^b^	171 (61)	50 (62)	121 (61)	11 (58)	160 (61)	0.425/0.078
Recipient- and operative characteristics						
Gender (F:M)	83 (30):198 (70)	27 (33):54 (67)	56 (28):	3 (16):16 (84)	80 (31):182 (69)	0.228/0.134
Age (years)	58 [51–63]	62 [57–66]	55 [48–62]	64 [57–67]	57 [51–62]	**0.000/0.001**
BMI	27 [24–31]	27 [24–30]	27 [24–31]	29 [24–32]	28 [25–31]	0.398/0.094
ALF	36 (13)	9 (11)	27 (14)	1 (5)	35 (13)	0.372/0.270
HCC	91 (32)	32 (40)	59(29)	10 (53)	81 (31)	0.070/**0.048**
Alcoholic cirrhosis	56 (29)	19(23)	37 (19)	5 (26)	51 (20)	0.217/0.321
Viral	20 (7)	2(2)	18(9)	0 (0)	20 (8)	**0.039**/0.234
PSC/PBC	19 (7)	3 (4)	16(8)	1 (5)	18 (7)	0.149/0.626
Other	59 (30)	16 (20)	43 (20)	2 (11)	57 (21)	0.536/0.267
Child–Pugh Score	7 [5–9]	7 [5–9]	7 [5–9]	7 [5–9]	7 [5–9]	0.630/0.786
labMELD	17 [10–27]	20 [10–27]	16 [9–27]	21 [14–25]	16 [10–27]	0.157/0.324
ALT pre-OLT (U/l)	41 [27–78]	31 [22–52]	44 [29–98]	31 [23–40]	42 [27–81]	**0.001/0.030**
AST pre-OLT (U/l)	58 [42–115]	45 [35–78]	63 [45–134]	54 [34–62]	58 [42–122]	**0.000**/0.159
GGT pre-OLT (U/l)	95 [51–220]	91 [41–199]	97 [55–238]	110 [59–233]	94 [51–215]	0.178/0.826
Bilirubin pre-OLT (mg/dl)	2.4 [1.0–9.5]	2.4 [0.8–7.6]	2.4 [1.1–10.3]	2.4 [1.1–4.3]	2.4 [1–10.3]	0.667/0.621
Pre-OLT ICU (days)	60 (20)	14 (17)	46 (23)	1 (5)	59 (23)	0.185/0.058
Pre-OLT abdominal surgery	92 (33)	34 (42)	58 (29)	5 (26)	87 (33)	**0.022**/0.362
Pre-OLT encephalopathy	107 (38)	34 (42)	73 (37)	4 (21)	103 (39)	0.212/0.085
Cold ischemic time (min)	490 [432–578]	497 [449–587]	490 [430–577]	528 [468–615]	484 [430–577]	0.543/0.171
Warm ischemic time (min)	45 [40–51]	45 [41–51]	46 [40–50]	42 [38–47]	46 [41–52]	0.428/0.058
Intra-operative RBC transfusions (units)	8 [4–12]	8 [4–13]	8 [4–12]	9 [7–13]	8 [4–12]	0.737/0.397
Intra-operative FFP transfusions (units)	16 [13–23]	17 [14–22]	16 [12–23]	20 [15–28]	16 [13–22]	0.380/0.096

Significant *p*-values (*p* < 0.05) are marked in bold

Abbreviations: *BMI* body mass index, *CVA* cerebrovascular accident, *ALF* acute liver failure, *HCC* hepatocellular carcinoma, *PSC* primary sclerosing cholangitis, *PBC* primary biliary cirrhosis, *labMELD* laboratory model for end-stage liver disease, *ALT* alanine aminotransferase, *OLT* orthotopic liver transplantation, *AST* aspartate aminotransferase, *GGT* gamma-glutamyl transferase, *ICU* intensive care unit, *RBC* red blood cell, *FFP* fresh frozen plasma, *CAC* celiac artery calcification, *VAC* visceral aortic calcification

^a^Based on Feng et al. [13]

^b^Based on German Medical Chamber guidelines [6]

### Calcification analysis

According to our predefined cutoffs, 87 (31%) of all patients showed relevant calcification of any of the regions of interest, from whom 81 (28.8%) had significant calcification of the visceral aortic segment (VAC) with a median Agatston score of 516 [211–805] mm^3^ and 19 (6.8%) of the celiac artery (CAC) with a median Agatston score of 115 [74–241] mm^3^. The AUROCs were 0.668 and 0.657, leading to the cutoff values of 100 mm^3^ and 50 mm^3^ (*J* = 0.331 and 0.278) for VAC and CAC, respectively.

Concerning demographics and clinical characteristics, patients with calcification of the visceral aortic segment or the celiac trunk were older (median age 62 vs. 55, *p* < 0.001; 64 vs. 57, *p* = 0.001, Table 1, respectively) than those without calcification. While labMELD did not differ significantly between the groups (20 [12–27] vs. 16 [9–27], *p* = 0.157; 21 [14–25] vs. 16 [10–27], *p* = 0.324, Table 1, respectively), patients with high VAC or CAC had lower ALT values prior to OLT (31 [22–52] vs. 44 [29–98], *p* = 0.001; 31 [23–40] vs. 42 [27–81], *p* = 0.03, Table 1, respectively) compared to patients without significant calcification. Concerning AST, lower values were found in VAC but not in CAC patients (45 [35–78] vs. 63 [45–134], *p* < 0.001; 54 [34–62] vs.42, 58 [42–122], *p* = 0.159, Table 1, respectively).

Chronic viral hepatitis was less frequent (2 vs. 9%, *p* = 0.039, Table 1, respectively) in patients with VAC, while patients with significant CAC showed a higher prevalence of hepatocellular carcinoma (53 vs. 31%, *p* = 0.048, Table 1, respectively). A higher number of patients with VAC had a history of pre-transplant abdominal surgery than those without VAC (42 vs 29%, *p* = 0.022, Table 1, respectively). Detailed patients’ characteristics are displayed in Table 1.

### Perioperative data and its association with VAC and CAC

**Fig. 2:**
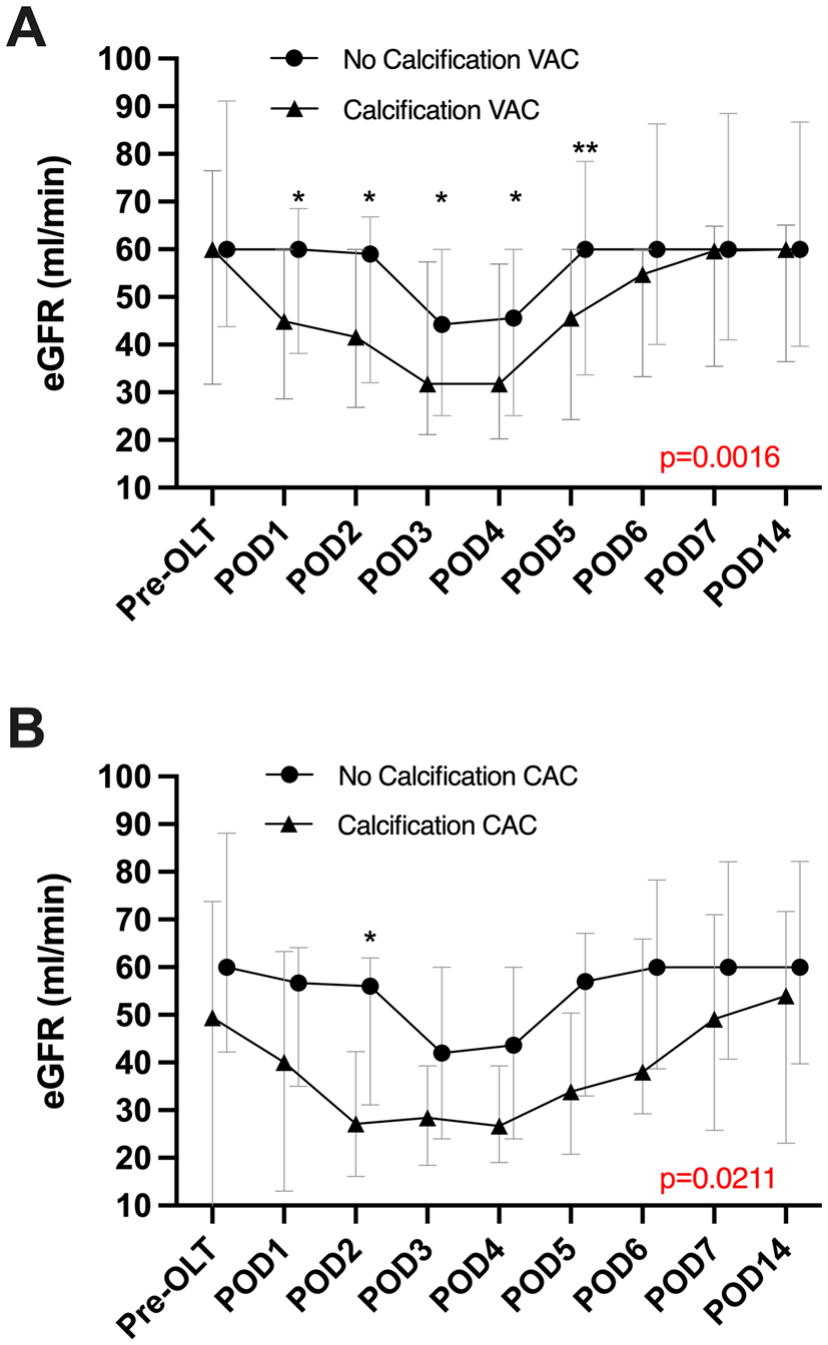
Estimated glomerular filtration rate pre-OLT and over the course of the first two weeks after liver transplantation. Patients receiving pre-OLT dialysis were excluded. Parameter stratified by critical calcification of the aorta (**a**) or the celiac artery (**b**). Data are shown as median and interquartile ranges (IQR). *OLT* orthotopic liver transplantation, *POD* postoperative day, *eGRF* estimated glomerular filtration rate, *CAC* celiac artery calcification, *VAC* visceral aortic calcification

**Fig. 3:**
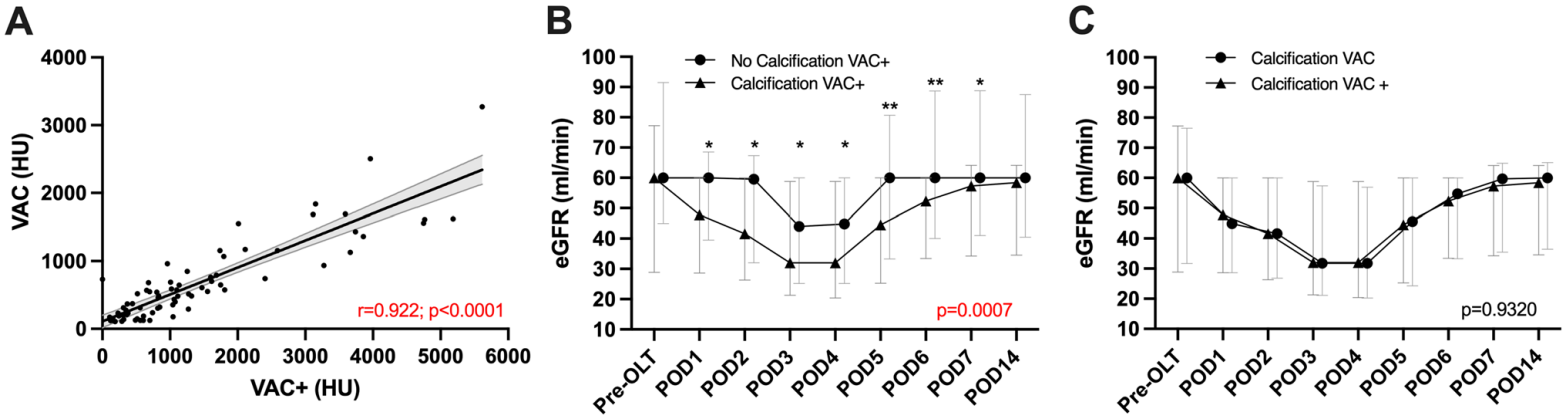
Results comparing VAC and VAC+ . **a** Correlation analysis between VAC and extended visceral aortic calcification values or VAC+ . Spearman correlation plot including ± 95% confidence interval. **b**, **c** Patients receiving pre-OLT dialysis were excluded. Estimated glomerular filtration rate stratified by VAC+ (**a**) and comparing VAC+ with VAC. Data are shown as median and interquartile ranges (IQR). *CAC* celiac artery calcification, *VAC* visceral aortic calcification, *VAC*+  extended visceral aortic calcifications including the renal artery ostia;, *HU*: Hounsfield units, *eGRF* estimated glomerular filtration rate

Patients with atherosclerotic involvement of either the visceral aorta or the celiac trunk showed a comparatively higher incidence of early postoperative renal function impairment, as determined by eGFR, compared to patients without calcifications in the first week following OLT (VAC: *p* = 0.0016; CAC: *p* = 0.0211; Fig. 2, respectively). In the analysis of kidney function, patients receiving pre-OLT dialysis were excluded (*n* = 42, 15%). Pre-OLT eGFR did not significantly differ between patients with significant calcification versus without (VAC: 60 [32–77] ml/min vs. 60 [44–91] ml/min *p* = 0.054; CAC: 60 [17–79] ml/min vs. 60 [42–88], *p* = 0.285; Fig. 3, respectively). Estimated GFR was prominently decreased with lowest values on the fourth postoperative day (32 [20–57] ml/min; Fig. 2, respectively) in patients suffering from VAC, while patients without aortic calcification showed worst renal function on POD 3 (44 [25–60] ml/min; Fig. 2, respectively). The most significant difference in renal function could be observed on POD 5 between the above-mentioned groups (46 [24–60] ml/min vs. 60 [34–78], *p* = 0.005; Fig. 3, respectively). Both, patients with and without CAC suffered the nadir of renal function impairment on POD 3 (28 [18–39] ml/min vs. 42 [24–60], *p* = 0.564; Fig. 2, respectively). Despite the continuous differences, observed graphically, the only statistically significant difference concerning eGFR levels was found on POD 2 (27 [16–42] ml/min vs. 56 [31–62], *p* = 0.029; Fig. 2, respectively).

Overall, severe postoperative complications (CD ≥ 3b) occurred in 56% of cases and the 90-day mortality after OLT was 9%. Neither of these parameters was significantly associated with visceral calcification (Table 2). Patients with significant VAC showed higher rates of EAD (38 vs. 26%, *p* = 0.031; Table 2, respectively), while patients with CAC did not (37 vs. 29%, *p* = 0.316; Table 2, respectively). While no statistically significant difference in estimated procedural costs was found between VAC and non-VAC patients (58 [40–92] TEuro vs 53 [39–80] TEuro, *p* = 0.284; Table 2, respectively), relevant CAC was significantly associated with higher procedural costs (70 [48–101] TEuro vs 53 [39–79] TEuro, *p* = 0.049; Table 2, respectively). Concerning length of ICU stay and length of overall hospitalization, no difference was found between the groups. The analysis of aortic and celiac trunk calcification levels and their association with perioperative outcome are shown in Table 2.

**Table 2:** Analysis of perioperative outcome based on the visceral aortic and celiac artery calcification cutoff

	All patients	VAC > 100 mm^3^		CAC > 50 mm^3^		*p* value
	*n* = 281	Yes *n* = 81	No *n* = 200	Yes *n* = 19	No *n* = 262	
90-day ≥ CD3b complications^a^ *n* (%)	156 (56)	47 (58)	109 (55)	14 (73)	142 (54)	0.343/0.077
90-day mortality *n* (%)	24 (9)	8 (10)	16 (8)	2 (11)	22 (8)	0.382/0.498
Early allograft dysfunction^b^ *n* (%)	82 (29)	31 (38)	51 (26)	7 (37)	75 (29)	**0.031/**0.316
Post-OLT RRT *n* (%)	72 (26)	19 (24)	53 (27)	6 (32)	66 (25)	0.356/0.353
ICU stay (days)	5 [3–11]	5 [3–11]	5 [3–11]	7 [4–13]	5 [3–11]	0.390/0.258
Hospital stay (days)	27 [20–50]	29 [20–55]	26 [19–47]	39 [19–53]	27 [20–49]	0.450/0.740
Post-OLT RBC transfusion (units)	2 [0–5]	2 [0–6]	2 [0–4]	2 [0–4]	2 [0–5]	0.522/0.970
Post-OLT FFP transfusion (units)	2 [0–7]	2 [0–8]	2 [0–6]	4 [0–8]	2 [0–6]	0.843/0.278
90-day CCI^c^	52 [43–87]	56 [30–98]	51 [35–81]	66 [47–100]	52 [32–82]	0.562/0.087
Cost estimation (TEuro)^d^	54 [40–85]	58 [40–92]	53 [39–80]	70 [48–101]	53 [39–79]	0.284/**0.049**

Significant *p*-values (*p* < 0.05) are marked in bold

Abbreviations: *CD* Clavien–Dindo classification, *OLT* orthotopic liver transplantation, *RRT* renal replacement therapy, *ICU* intensive care unit, *RBC* red blood cell units, *FFP* fresh frozen plasma units, *CCI* Comprehensive Complication Index, *TEuro* thousand Euros, *CAC* celiac artery calcification, *VAC* visceral aortic calcification

^a^Based on Clavien et al. [24]

^b^Based on Olthoff et al. [22]

^c^Based on Slankamenac et al. [25]

^d^Based on Staiger et al. [27]

Finally, in the uni- and multivariable logistic regression analyses (Table 3), recipient age (0.634 OR, 0.239–1.089 CI, *p* = 0.007; Table 3, respectively), cold ischemic time (1.931 OR, 1.115–3.346 CI, *p* = 0.019; Table 3, respectively), and VAC (2.387 OR, 1.290–4.418 CI, *p* = 0.006; Table 3, respectively) were identified as independent predictors of EAD.

**Table 3:** Uni- and multivariable logistic regression analyses for early allograft dysfunction

	EAD^a^ *n* = 82	No EAD^a^ *n* = 199	Univariable analysis		Multivariable analysis	
			Odds ratio (95% Confidence Interval)	*p value	Odds ratio (95% Confidence Interval)	*p* value
Donor age ≥ 65 years	11 (13)	47 (24)	0.673 (0.378–1.199)	0.179		
Donor BMI ≥ 25	66 (81)	150 (77)	1.320 (0.688–2.534)	0.404		
Donor Sex Male	40 (49)	102 (52)	0.868 (0.518 -1.455)	0.592		
Pre-transplant Child–Pugh Score ≥ 7	38 (46)	98 (50)	0.946 (0.431–2.075)	0.889		
ECD^b^ Yes	54 (66)	114 (59)	1.588 (0.845–2.985)	0.151		
Recipient age ≥ 60 years	27 (33)	86 (44)	**0.634 (0.369–1.089)**	**0.099**	**0.5437 (0.239–0.799)**	**0.007**
Recipient BMI ≥ 25	59 (72)	122 (63)	1.535 (0.875–2.693)	0.135		
Recipient Sex Male	59 (72)	135 (69)	1.140 (0.645–2.015)	0.652		
Pre-transplant labMELD ≥ 25	29 (35)	55 (28)	1.535 (0.875–2.693)	0.135		
Recipient pre-transplant ICU Yes	22 (27)	37 (19)	1.566 (0.854–2.869)	0.147		
Recipient pre-transplant abdominal surgery Yes	32 (39)	58 (30)	1.543 (0.898–2.650)	0.116		
Recipient pre-transplant encephalopathy Yes	32 (39)	74 (38)	1.068 (0.628–1.816)	0.809		
Cold ischemic time ≥ 540 (min)	38 (46)	67 (34)	**1.649 (0.973–2.795)**	**0.063**	**1.931 (1.115–3.346)**	**0.019**
Warm ischemic time ≥ 45 min	48 (59)	112 (58)	1.026 (0.605–1.741)	0.924		
Visceral Aortic Calcification Yes:	31 (38)	50 (26)	**1.763 (1.017–3.055)**	**0.043**	**2.387 (1.290–4.418)**	**0.006**
Celiac Artery Calcification Yes:	7 (9)	12 (6)	1.423 (0.540–3.755)	0.476		

Significant odds-ratios and *p*-values (*p* < 0.05) are marked in bold

Abbreviations: *BMI* body mass index, *ECD* extended criteria donor allografts, *labMELD* laboratory model for end-stage liver disease, *ICU* intensive care unit

Values were given as median [interquartile ranges (IQR)] or numbers and (per cent). Results of the logistic regression were given as odds ratios with 95% confidence interval

^a^According to Olthoff et. al. [7]

^b^Based on the German Medical Chamber Guidelines [6]

*Factors showing a *p* value < 0.1 in the univariable analysis were included in the multivariable logistic regression model

### *VAC and VAC*+ 

As VAC has been shown to have a significant impact on renal outcomes, as a subsequent step VAC+ has been introduced, involving a secondary measurement with the inclusion of both renal artery ostia (Fig. 1). VAC+ has demonstrated a similar AUROC and Youden index (0.623, *J* = 0.298) like VAC, yielding a cutoff value of 200 mm^3^. A total of 85 patients (30%) suffered from a significant level of calcification based on VAC+. 

VAC and VAC+ have shown a very strong association (*r* = 0.922; *p* < 0.0001, Fig. 3). Renal outcomes showed a largely similar pattern like with VAC and eGFR values of patients with critical calcification based on VAC versus VAC+  were nearly identical at each time point (Fig. 3). Further perioperative data including complications, hospital stay, transfusions were almost identical between VAC+ and VAC patients (Supplementary Table 1).

Therefore, the extended measurement of VAC+ has failed to provide a significantly superior value in assessing perioperative outcomes compared to VAC.

### Long-term graft and patient survival

**Fig. 4:**
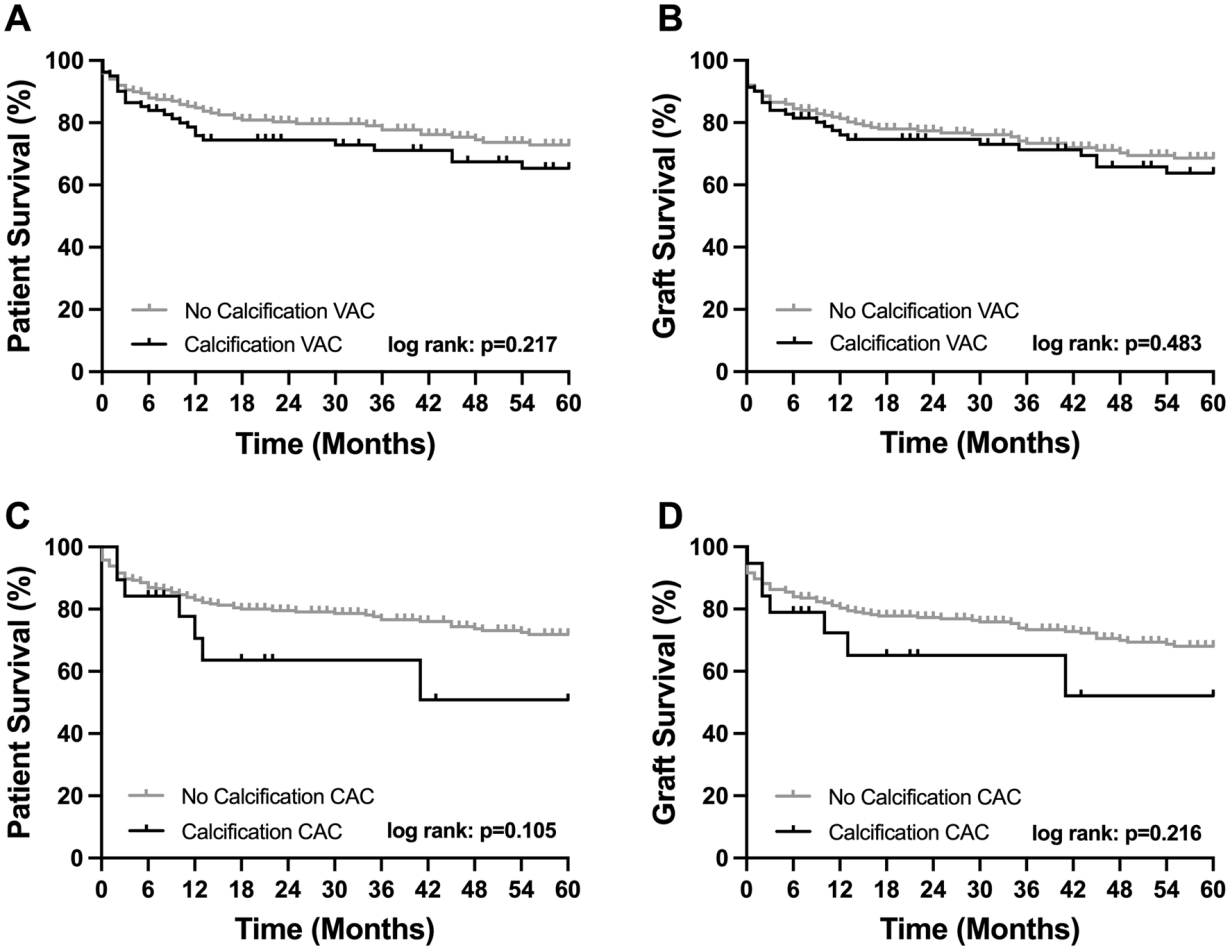
Graft and patient survival stratified by significant calcification of the visceral aorta and celiac artery. **a** 5-year patient and **b** graft survival by critical calcification of the visceral aorta (64%) vs. no significant calcification (69%). **c** 5-year patient and **d** graft survival by critical calcification of the celiac artery (52%) vs. no significant calcification (68%). *CAC* celiac artery calcification, *VAC* visceral aortic calcification

Patients who died within the first 90 days following OLT (*n* = 24) were not excluded from this analysis as no significant effects of VAC or CAC on short-term mortality were detected (Table 2, respectively). The median length of follow-up for the patient cohort was 70 months. Calcification of neither the visceral aorta nor the celiac trunk had significant effects on long-term graft and patient survival. Even though not statistically significant, high levels of VAC showed slightly inferior graft (1 year: 76 vs. 81%, 3 years: 71 vs. 73%, 5 years: 64 vs. 69%, *p* = 0.483; Fig. 4, respectively) and patient survival rates (1 year: 76 vs. 85%, 3 years: 71 vs. 78%, 5 years: 65 vs. 73%, *p* = 0.217; Fig. 4, respectively). In line with these findings, graft (1 year: 71 vs. 81%, 3 years: 65 vs. 73%, 5 years: 52 vs. 68%, *p* = 0.216; Fig. 4, respectively) and patient survival (1 year: 71 vs. 83%, 3 years: 65 vs. 80%, 5 years: 52 vs. 72%, *p* = 0.105; Fig. 4, respectively) were slightly inferior in patients with CAC, even though this difference did not reach the level of statistical significance (Fig. 4).

## Discussion

Currently clinical risk assessment in OLT is predominantly based on the subjective clinical judgment of a senior physician. Despite major improvements of the recent decades in surgical methods, organ preservation, intensive care treatment, and immunosuppression, OLT is still associated with high morbidity and mortality. In this context, cardiovascular disease is known to be the leading cause of non-graft-related mortality following OLT [31]. Recent results of Roehl et al. underlined the importance of preoperative calcification analysis in patients scheduled for OLT in terms of cardiovascular outcomes [2]. In combination with Lee’s revised cardiac index, the authors could demonstrate the high sensitivity of coronary artery calcification in pre-OLT CT scan in identifying patients at risk for cardiovascular events after transplantation [4].

Although several studies showed impaired postoperative outcomes including higher incidence of anastomotic leakage in patients with significant atherosclerosis undergoing major gastrointestinal surgery [32, 33], there is still a lack of data on its role in patients undergoing deceased donor OLT. While Imaoka et al. recently reported that VAC is associated with impaired long-term survival focusing mainly on patients after living donor liver transplantation [19], the present study is, to the best of our knowledge, the first report on the clinical implications of VAC and CAC in the deceased donor OLT setting assessing morbidity and mortality and long-term outcomes.

The present analysis has led to some important observations. In terms of perioperative outcomes, we could register a higher rate of EAD in patients with significant aortic calcification (VAC) compared to the rest of the cohort. Meanwhile patients with CAC showed a tendency towards worse outcome in terms of 90-day morbidity with a marginally non-significant difference in major complications (Clavien–Dindo ≥ CD3b) and CCI. Both CAC and VAC patients have shown a significant (VAC: *p* = 0.0016; CAC: *p* = 0.0211) impairment in the early perioperative kidney function (eGFR). However, VAC patients have also presented with a worse pre-OLT eGFR. Both curves have reached their pre-OLT baseline after approximately 14 days following OLT, suggesting only a temporary effect on renal outcomes.

Although this has not been investigated in the setting in OLT before, the observation is in line with previous studies showing an association of VAC with a decline in GFR in general [34].

On the level of the underlying pathophysiology, this phenomenon might be explained by the fact that high calcification levels promote stiffness of the vessels resulting in isolated systolic hypertension which causes target organ damage [35, 36]. This hypertension is accompanied by normal or even low diastolic pressure which promotes enhanced pulsatile energy penetration into the microvasculature of the organs including the kidneys, which must therefore operate at low arteriolar resistance [37]. This low resistance and high blood flow expose the kidneys to pulsatile pressure and blood flow stress, which can damage the glomeruli, resulting in a lower glomerular filtration rate and less functional renal reserves [37, 38]. Additionally, it has to be taken into consideration that declines in renal function are associated with factors including dysregulation of bone and mineral metabolism and increased serum levels of various inflammatory cytokines [39, 40]. These dysregulations of bone and mineral metabolism combined with a pro-inflammatory state are known to further worsen arteriosclerosis resulting in an increasing stiffness of the aorta, which in turn promotes renal function loss, initially on a subclinical and later on a clinical level [36]. In line with this, VAC levels are in general higher in patients suffering from end-stage renal disease compared to those with normal kidney function or vice versa [41]. In this study, the temporary nature of the renal function impairment following OLT might be explained by an acute on chronic injury, due to hypovolemia, perioperative relative ischemia, inflammation, drug toxicity combined with a previous kidney damage and reduced functional reserve capacity [42].

A further important observation of this study was that the tendentially higher morbidity seen in CAC patients translated into 17.000 € higher estimated median costs over the first 90 days following OLT. This is an important finding showing the direct health-economical relevance when transplanting these high-risk patients.

In terms of perioperative outcomes, Imaoka et al. have observed a higher rate of blood stream infections in the high VAC group compared to the rest of the patients in a Japanese cohort of 156 consecutive liver transplantation [19].

As an additional result, VAC has been shown to be an independent risk factor for EAD in the multivariate analysis with a meaningful OR of 2.387. Post-OLT EAD is associated with increased length of hospitalization, higher procedural costs, and impaired short- and long-term graft survival in earlier studies [43, 44]. In order to improve the outcome of OLT, strategies to lower the incidence of EAD are required. Although it requires further validation and remains speculative, based on this data we can assume that patients with advanced VAC might benefit from balancing their overall EAD risk by accepting higher quality allografts or organs with shorter cold ischemia times.

It should be noted as well that the literature is largely heterogeneous concerning the anatomical landmarks and measurement approaches to quantify calcification of the visceral arteries. Some studies are only analyzing the proximal visceral segment to define VAC, while others are focusing on the whole abdominal aorta [19]. There is no international consensus or recommendation on how this should be approached. Our group decided to focus on the proximal part of the abdominal aorta based on three considerations: (I) Assuming that the proximal segment will have the most significant hemodynamical and clinical relevance in the OLT setting as the celiac trunk is responsible for the arterial supply of the liver and large part of the upper abdomen. (II) In our institutional routine, most OLT patients only receive a pre-transplant upper abdomen scan which usually does not include the very distal part of the aorta (e.g., aortic bifurcation). (III) A further aspect was the possibility of a broad utilization and potential further validation. Some groups are even more restrictive concerning their standards in abdominal imaging, as they are only performing CT scans focusing on the vascular anatomy of the liver itself where often not even the renal artery ostia are fully depicted. However, to further explore the observed difference in renal function, we have carried out a secondary VAC measurement where both ostia of the renal arteries were included (VAC+). In this secondary comparative analysis, VAC and VAC+ values have shown a strong correlation and VAC+ has failed to provide a further significant benefits over VAC in the stratification of patients in terms of renal function and other perioperative outcomes. Although this needs further validation, our data suggest that VAC and VAC+ can be used interchangeably, depending on the availability of the distal aortic segments in the scan.

Last but now least we have assessed long-term graft and patient survival stratified by VAC and CAC in our cohort. Here we could observe a graphically slightly worse outcome in the calcified groups; however, due to the limited sample size and statistical power, these differences have not reached the level of statistical significance. This is in contrast to the findings of Imaoka et al. who could show a significantly worse (*p* = 0.004) patient survival in the high VAC group [19].

The findings of this study should be interpreted in the light of possible limitations. First, the retrospective, single-center nature of this study is one of its most important limitations. Especially, the small cohort of patients suffering from significant CAC results in limited power of our statistical analysis when interpreting between-group differences in terms of clinical outcomes such as EAD, complications, and survival. Second, our patient population exhibits the features of a heterogeneous European OLT patient cohort, which may result in underrepresentation of various indications and patient subgroups due to selection bias (e.g., high-MELD patients or patients with NASH). Third, due to the physiological evolution of atherosclerosis with age, recipients suffering from calcification were significantly older presenting an important cofounder in this analysis. Likewise, the full complexity of the overall risk in the OLT setting with a number of donor, recipient, and (peri)operative factors potentially negatively influencing outcomes could not be depicted here which is a general limitation to any similar conventional statistical model. Therefore, multiple confounding factors were ignored (e.g., pre-existing comorbidities including hypertension, heart failure or hypercholesterolemia, donor vascular status, hypotensive periods during the perioperative phase). A properly designed machine learning model and artificial intelligence-driven registration of perioperative clinical data might be an answer for this limitation in the future, integrating, weighing, and modeling the effect of a large number of factors at the same time [45, 46]. In addition, the evaluation of calcification and therefore the division into significant and non-significant calcification groups were performed solely with respect to plaque quantification. Further on, some patients exhibit mixed atheromatous and calcified atherosclerosis, whereas calcium scoring using the Agatston method only detects calcified plaques due to its density cutoffs. This leaves out the effect of soft plaques, which may be prevalent in up to 60% of atherosclerotic lesions of the abdominal aorta [47].

Despite the aforementioned limitations, the current study used well-established and robust statistical methods to describe the clinical importance of visceral calcium quantification in pre-OLT CT-scans. Further strengths of our study are the careful assessment of 90-day postoperative complications according to Clavien–Dindo and the utilization of the CCI score, reporting cost-analysis data as well as the large overall cohort of patients and the long follow-up period.

## Conclusions

The benefit of calcification scoring lies in its simplicity and broad accessibility of preoperative CT scans for assessment. Future studies should focus on the specific mechanism behind the negative prognostic effects of VAC and CAC as well as on establishing any association with overall survival and graft loss. Although the level of calcification will presumably never be able to replace the risk assessment and subjective judgment concerning "suitability for transplant" by an experienced transplant physician, it might be a valuable additional clinical tool with certain limitations.

## Supplementary Information

Below is the link to the electronic supplementary material.

Supplementary Table 1 (PDF 45 KB)

## Data Availability

All relevant data were reported within the manuscript and the supplementary files. Further supporting data will be provided upon written request addressed to the corresponding author.

## Abbreviations

ALF: Acute liver failure
ANOVA: Analysis of variance
BAR: Balance of risk
BC: Body composition
BMI: Body mass index
CAC: Celiac artery calcification
CCI: Comprehensive complication index
CD: Clavien–Dindo classification
CT: Computed tomography
CVA: Cerebrovascular accident
DRI: Donor risk index
EAD: Early allograft dysfunction
EASE: Early allograft failure simplified estimation
ECD: Extended criteria donor
eGFR: Estimates glomerular filtration rate
FFP units: Fresh frozen plasma units
GCP: Good clinical practice
ICU: Intensive care unit
L-GrAFT: Liver Graft Assessment Following Transplantation
MELD: Model of end-stage liver disease
OLT: Orthotopic liver transplantation
OR: Odds ratio
PBC: Primary biliary cholangitis
POD: Postoperative day
PSC: Primary sclerosing cholangitis
RBC units: Red blood cell units
SOFT: Survival outcomes following liver transplantation
TEur: Thousand Euros
VAC: Visceral aortic calcification
VAC +: Extended visceral aortic calcification
UH-RWTH: University Hospital RWTH Aachen
