# Supplementary material for: Calcification of the visceral aorta and celiac trunk is associated with renal and allograft outcomes after deceased donor liver transplantation
Source: Abdom Radiol (NY). 2022 Nov 28;48(2):608–20. doi: 10.1007/s00261-022-03629-8 (PMC9902327; doi:10.1007/s00261-022-03629-8)
Supplement: Supplementary file 1 — Supplementary Table 1 (PDF 45 KB) [file 261_2022_3629_MOESM1_ESM.pdf]

**Supplementary Table 1:** Perioperative outcome comparing visceral aortic (VAC) and extended visceral aortic calcification (VAC+)

| | All patients | VAC | VAC+ > 200 mm³ | | p-value |
|---|---|---|---|---|---|
| | **n=281** | **yes=81** | **yes n=85** | **no n=196** | |
| **90-day ≥CD3b complications[a] n (%)** | 156 (56) | 47 (58) | 53 (62) | 103 (53) | 0.358 |
| **90-day mortality n (%)** | 24 (9) | 8 (10) | 10 (12) | 14 (7) | 0.225 |
| **Early allograft dysfunction[b] n (%)** | 82 (29) | 31 (38) | 34 (40) | 48 (25) | **0.041** |
| **Post-OLT RRT n (%)** | 72 (26) | 19 (24) | 20 (24) | 52 (26) | 0.386 |
| **ICU stay (days)** | 5 [3-11] | 5 [3-11] | 5 [3-11] | 5 [3-11] | 0.412 |
| **Hospital stay (days)** | 27 [20-50] | 29 [20-55] | 29 [20-55] | 26 [20-47] | 0.689 |
| **Post-OLT RBC transfusion (units)** | 2 [0-5] | 2 [0-6] | 2 [0-6] | 2 [0-4] | 0.985 |
| **Post-OLT FFP transfusion (units)** | 2 [0-7] | 2 [0-8] | 2 [0-8] | 2 [0-6] | 0.612 |
| **90-day CCI[c]** | 52 [43-87] | 56 [30-98] | 54 [30-89] | 52 [35-84] | 0.903 |
| **Cost estimation (TEuro)[d]** | 54 [40-85] | 58 [40-92] | 55 [40-92] | 53 [40-801] | 0.431 |

[a]Based on Clavien et al. 2009. [b]Based on Olthoff et al. 2010. [c]Based on Slankamenac et al. 2013. [d]Based on Staiger et al. 2018. Abbreviations used: VAC: visceral aortic calcification; VAC+: extended visceral aortic calcification; CD: Clavien-Dindo classification; OLT: orthotopic liver transplantation; RRT: renal replacement therapy; ICU: intensive care unit; RBC: red blood cell units; FFP: fresh frozen plasma units; CCI: Comprehensive Complication Index; TEuro: thousand Euros
